# Deposition characteristics of a new allergic rhinitis nasal spray (MP29-02*) in an anatomical model of the human nasal cavity

**DOI:** 10.1186/2045-7022-5-S4-P40

**Published:** 2015-06-26

**Authors:** Alex D’Addio, Nancy Ruiz, Michael Mayer, Ruth Murray, Claus Bachert

**Affiliations:** 1 Meda, Somerset, NJ, USA; 2 Next Breath LLC, Baltimore, MD, USA; 3 Medscript Ltd, Dundalk, Ireland; 4 Ghent University Hospital, Department of Oto-Rhinolaryngology, Ghent, Belgium

## Background

Intranasal sprays must be delivered to the nasal cavity in sufficient volume, appropriate viscosity and droplet size and with a technique that allows optimal retention, maximizes absorption from the mucosa, and the potential for maximum therapeutic effect. The aim of this study was to evaluate nasal drug run-off after administration of MP29-02* (a novel intranasal formulation of azelastine hydrochloride (AZE) and fluticasone propionate (FP) in an advanced delivery system) with sequential administration of marketed AZE and FP nasal sprays in vitro.

## Methods

A normal adult human nasal cavity in vitro model was used [1,2]. A single spray of MP29-02* (0.137 mL [137µg AZE/50µg FP]) or single sequential sprays of AZE (0.137 mL) followed 1 min later by either branded or generic FP (0.100 mL; i.e. multiple therapy) were manually actuated into the model (away from the septum). A slight vacuum was applied during spray delivery to simulate inhalation.

## Results

Three replicates of MP29-02* showed no dripping or back flow from the nasal cavity (i.e. anterior spray area or anterior drip = 0.00 cm2). In all replicates MP29-02* was observed to coat all turbinates up to the nasopharynx, but not the nasopharynx structure itself. However, three replicates of sequential sprays of AZE followed 1 min later by either branded or generic FP showed significant anterior nasal drip (i.e. run-off) from the nostril and also toward the back of the nasal cavity (i.e. posteriorly, which would be swallowed in vivo); AZE & branded FP: anterior spray area = 1.67 – 3.16 cm2; AZE & generic FP: anterior spray area: 0.68 – 1.83 cm2.

## Conclusion

MP29-02* is a new AR treatment, comprising AZE and FP in a single spray in an improved formulation and device (vs marketed FP). In this model, the delivery of MP29-02* showed improved retention in the targeted areas compared to sequential administration of marketed intranasal monoproducts. These could not be administered together without run off (posteriorly and anteriorly) which could diminish efficacy.

## 

* Dymista
